# Navigating uncertainty: The impact of environmental instability on enterprise digital transformation

**DOI:** 10.1371/journal.pone.0314688

**Published:** 2024-12-05

**Authors:** Feifei Luo, Chia-Hsien Tang

**Affiliations:** Economics and Management Department, Guangxi Minzu Normal University, Chongzuo, Guangxi, China; University of Innsbruck: Universitat Innsbruck, AUSTRIA

## Abstract

This study investigates the impact of environmental uncertainty on the digital transformation of Chinese A-share listed companies from 2013 to 2022. Our empirical analysis reveals that environmental uncertainty negatively affects digital transformation by inducing managerial myopia, hindering research and development (R&D) investment, and exacerbating financial constraints. However, these negative effects can be mitigated by increasing management shareholding, which aligns managers’ interests with long-term goals, providing digital transformation subsidies to lower financial barriers, and fostering a favorable legal environment to protect digital investments and innovation. These findings offer valuable insights for understanding how environmental uncertainty influences digital transformation strategies and provide practical implications for enterprises and policymakers in dynamic business environments.

## Introduction

The rapid advancement of digital technologies such as big data, artificial intelligence, cloud computing, and blockchain is revolutionizing industrial processes, fostering economic transitions, and driving high-quality growth in China [1,2]. China’s "Overall Layout Plan for Digital China" (2023) underscores the nation’s strategic focus on cultivating core digital industries, enhancing the digital economy, and fostering deep integration between digital technology and the real economy [3]. Enterprises, as key economic players, are actively leveraging these cutting-edge technologies to embark on digital transformation journeys. This transformation enables firms to activate data as a new production factor, enhance value creation through technological empowerment, improve productivity, bolster risk resilience, and ultimately achieve sustainable growth [4].

Nevertheless, the road to digital transformation is fraught with challenges. According to accenture’s 2023 report, most Chinese enterprises are still navigating the early stages of this process [5]. In addition to grappling with high costs, capability gaps, and the disruptive nature of technological integration, firms are also confronted with a high volatile and uncertain external operating environment. This environmental uncertainty is driven by a confluence of factors such as macroeconomic policy shifts, industrial restructuring, public health crises, geopolitical conflicts, and unpredictable market dynamics [6]. Global events like the economic downturn, the COVID-19 pandemic, the Russia-Ukraine conflict, and increasing trade protectionism have further intensified these uncertainties, posing significant challenges for Chinese enterprises engaged in digital transformation [4,7].

In this study, we we adopt a comprehensive definition of environmental instability, which we define as the volatility or unpredictability of a firm’s external operating environment. Environmental instability encompasses a wide range of factors beyond policy-related uncertainties, such as macroeconomic fluctuations, industry-specific shocks, natural disasters, technological disruptions, and geopolitical tensions. Unlike economic policy uncertainty (EPU), which focuses narrowly on uncertainties related to government policies and regulations, environmental instability captures the broader spectrum of external shocks that may affect firms’ strategic decisions, including their digital transformation efforts.

Prior research has largely focused on the influence of corporate governance, managerial experience, government incentives, tax policies, and industry peer effects on digital transformation [6]. Although some studies suggest that economic policy uncertainty may encourage firms to adopt digital technologies as a form of risk mitigation [8], others contend that environmental shocks might hinder such investments by exacerbating financial instability [2]. While EPU plays an important role in the external environment, it represents just one facet of the uncertainties firms face. Our study expands this perspective by examining a more comprehensive range of environmental factors that influence firms’ digital transformation decisions. These include, but are not limited to, policy-related uncertainties, macroeconomic fluctuations, industry-specific disruptions, and unforeseen external events.

Despite the insights gleaned from previous studies, there remains a significant gap in understanding the direct impact of environmental uncertainty on enterprise digital transformation. Existing literature has yet to fully articulate the mechanisms through which these uncertainties influence firms’ digital transformation strategies. Additionally, much of the research relies on indirect inferences rather than direct empirical investigation, leaving room for further exploration.

To address this gap, our study empirically examines the impact of environmental instability measured through firm-level performance fluctuations on digital transformation among Chinese enterprises. We delve into the underlying mechanisms by which environmental instability influences these transformations and investigate the moderating roles of management shareholding, government subsidies, and the legal environment. By exploring these dynamics, our research offers valuable insights for firms and policymakers as they navigate the complexities of digital transformation in an increasingly uncertain world. We also aim to provide a theoretical foundation for government policies designed to support enterprise digital transformation, while offering practical guidance to firms seeking to thrive in volatile environments.

The paper is structured as follows: Literature and hypothesis development section reviews the relevant literature. Methods section explians the methodology, data, and model. Empirical section presents the empirical results. Disscussion and conclusion sections concludes with a discussion of the findings, limitations, and avenues for future research.

## Literature and hypothesis development

### Literature review

Firms today navigate environments that are not only dynamic but also inherently unpredictable, with uncertainty serving as a constant force shaping their strategic choices. [9] observed that environmental uncertainty arises when decision-makers lack sufficient information about external conditions, hindering their ability to anticipate outcomes or predict how these factors might influence their decisions. To capture this volatility, many studies quantify environmental uncertainty using the standard deviation of a firm’s revenue over several years, adjusted for industry-specific contexts [10].

Interestingly, research indicates that environmental uncertainty can have both stimulating and inhibiting effects on firms’ decision-making. On one hand, uncertainty can act as a catalyst for innovation, prompting decision-makers to confront risks head-on by accelerating technological progress [11]. On the flip side, heightened uncertainty can lead to cautious behavior, with some scholars arguing that the elevated risk of failure dampens firms’ enthusiasm for innovation, encouraging a more conservative approach [12]. Moreover, environmental uncertainty tends to increase firms’ capital costs by exacerbating information asymmetry with investors, which in turn raises the cost of equity [13]. These uncertain conditions also prompt auditors to issue more non-standard opinions, seeking to mitigate potential liability [14].

In response to these volatile environments, digital transformation has emerged as a critical strategy for firms. It involves integrating advanced technologies such as artificial intelligence, big data analytics, and the Internet of Things into a firm’s operations. This transformation is viewed as essential for enhancing organizational resilience and adaptability. Scholars have identified a variety of factors influencing digital transformation at both micro and macro levels. At the micro level, managers with R&D backgrounds and CEOs with IT expertise are better positioned to lead digital initiatives, using their knowledge to overcome resistance and drive innovation [15]. Additionally, human capital factors like employee education and skills have been shown to enhance innovation capacity and reduce managerial short-termism, supporting firms’ digital transformation efforts [7]. Strong corporate governance and dynamic capabilities are also crucial in driving digital initiatives within organizations [16].

At the macro level, government policies and incentives play an equally significant role in promoting digital transformation. Government subsidies, tax incentives, and robust digital infrastructure help alleviate financing constraints, reduce information asymmetry, and foster collaboration between industries and research institutions, thereby facilitating the adoption of new technologies [17,18]. Moreover, digital transformation efforts within an industry often generate positive spillover effects, driving convergence in digitization levels across firms [14].

### The impact of environmental uncertainty on digital transformation: Competing perspectives

Risk preference theory posits that most economic agents tend to display risk aversion when engaging in decision-making processes [11]. This aversion is amplified when facing external environmental instability, which disrupts established market expectations and amplifies operational risks [9]. Consequently, firms may hesitate in pursuing digital transformation initiatives due to the inherent uncertainties and potential negative consequences involved [19,20]. This hesitation stems from the fear of committing resources to potentially irreversible investments in a volatile environment, where the desired outcomes are less certain [21]. As [22] highlight, decision-makers might opt for a more cautious "wait-and-see" approach, delaying or even avoiding digital investments until the external situation stabilizes.

Empirical evidence supports this cautious approach. For instance, [3] found that environmental uncertainty negatively influences companies’ willingness to adopt new digital technologies, mainly because of the risks tied to volatile market conditions. Similarly, [23] observed that firms may adjust or postpone their digital innovation strategies in response to increased environmental uncertainty, primarily to mitigate the risks of financial loss and technological obsolescence. This cautiousness is further exacerbated by the growing demands for specialized skills associated with digital technologies, which may intensify anxieties surrounding job security and competency among employees, potentially fostering resistance and diminishing the effectiveness of transformation efforts [24–26].

On the other hand, growth option theory offers a contrasting viewpoint, suggesting that investment decisions should not be solely based on immediate cash flow considerations but also on long-term growth potential [27]. From this perspective, environmental instability can actually act as a catalyst for digital transformation. By embracing digital technologies, firms can enhance their ability to monitor and adapt to external changes, mitigating risks while increasing resilience [28]. Furthermore, digital transformation can result in enhanced operational efficiencies, superior product quality, and the discovery of new market opportunities via data-driven insights [29,30].

However, the applicability of growth option theory may be limited in environments characterized by extreme instability. The significant costs and complexities involved in digital transformation, along with the risk of technological obsolescence and organizational disruption, may ultimately outweigh the expected future benefits [31]. As [14] emphasize, environmental uncertainty could serve as a substantial barrier to digital transformation, particularly for firms with constrained resources or inadequate resource orchestration strategies. Similarly, the dynamic capabilities framework highlights the need for firms to reconfigure their resources and capabilities in response to shifting external conditions [32]. In such volatile contexts, companies might prioritize flexibility and resource conservation, leading them to either delay or scale back digital transformation efforts that require significant investment and organizational restructuring [33].

While various theoretical frameworks provide divergent perspectives, the majority of evidence derived from risk aversion theory, real options theory, and dynamic capabilities theory suggests that environmental instability is more likely to impede digital transformation than to promote it. The potential drawbacks of making irreversible digital investments in unstable environments, coupled with the necessity of preserving flexibility and conserving resources, may overshadow the projected benefits. Thus, we propose the following hypothesis:

Hypothesis 1:Environmental uncertainty inhibits enterprise digital transformation.

### Mechanisms affecting digital transformation

Managerial myopia, which refers to a focus on short-term gains at the expense of long-term value creation, is a well-documented phenomenon in corporate decision-making [34]. According to agency theory, environmental uncertainty can exacerbate this tendency due to heightened information asymmetry and agency costs. In uncertainty environments, managers may prioritize actions that secure short-term survival, such as cutting costs, hoarding cash, or pursuing projects with quick payoffs, often at the expense of long-term strategic initiatives like digital transformation [35]. Digital transformation, with its long investment horizons and uncertain returns, is frequently misaligned with short-term managerial objectives. Managers under external pressure to deliver immediate results may be reluctant to allocate resources to digital initiatives that are unlikely to yield immediate payoffs, further hindering digital transformation efforts. This misalignment between managers’ short-term priorities and shareholders’ long-term interests can stifle investment in digital technologies. Therefore, we propose:

Hypothesis 2: Environmental uncertainty inhibits enterprises’ digital transformation by increasing managerial myopia.

Research and development (R&D) serves as a critical driver of innovation and long-term growth for firms. However, environmental uncertainty can create a disincentive for R&D investment, particularly in digital technologies that are often characterized by high risk and uncertainty. The unpredictable nature of market conditions, technological advancements, and regulatory shifts can erode the expected returns on R&D investments, making them less attractive to risk-averse firms [36].

Moreover, knowledge spillover effects in digital technologies may amplify this problem. Firms may fear that competitors will quickly imitate their innovations, thereby reducing the competitive advantage derived from R&D. This fear is heightened in uncertain environments, where the risk is perceived to be greater. Consequently, firms may reduce their R&D activities, which in turn impedes their ability to adopt and implement digital technologies, ultimately hindering digital transformation [37]. Thus, we hypothesize:

Hypothesis 3: Environmental uncertainty inhibits enterprises’ digital transformation by weakening the intensity of enterprises’ R&D investment.

Financial constraints, defined as limitations on a firm’s access to external financing, can significantly impede digital transformation efforts. In uncertain environments, these constraints can become more pronounced. Heightened uncertainty can undermine investor confidence, leading to reduced access to capital markets and tighter lending conditions. At the same time, economic instability can negatively impact firms’ cash flows and profitability, further limiting their internal resources for digital investments [38].

Additionally, lenders may perceive firms as being at higher risk of default during periods of instability, leading to increased borrowing costs and more stringent lending conditions [39]. This make borrowing more costly and less attractive, particularly for long-term investments like digital transformation that may not yield immediate returns. These financial constraints can force firms to prioritize short-term survival over long-term strategic investments, such as digital transformation, which may not generate immediate returns. Therefore, we propose the following hypothesis:

Hypothesis 4: Environmental uncertainty inhibits enterprises’ digital transformation by increasing corporate financing constraints.

### Moderating the negative impact of environmental uncertainty on digital transformation

The preceding analysis has established that environmental uncertainty can act as a deterrent to digital transformation, primarily by amplifying managerial short-sightedness, discouraging R&D investments, and intensifying financial constraints. However, firms are not passive recipients of these challenges. They can proactively implement internal mechanisms and leverage external support systems to mitigate the adverse effects of uncertainty. In this context, we explore three potential moderators that could buffer the negative relationship between environmental uncertainty and digital transformation: increased management shareholding, government subsidies for digital transformation, and a favorable legal environment.

### Management shareholding

Agency theory posits that aligning the interests of managers with those of shareholders can reduce opportunistic behavior and promote long-term value creation [8]. In the context of digital transformation, increasing management shareholding can serve as a powerful incentive mechanism, encouraging managers to adopt a long-term perspective even amidst environmental uncertainty [25]. By holding a stake in the company, managers become more invested in its future success and are less likely to prioritize short-term gains at the expense of strategic digital initiatives. The increased personal cost of short-sighted decisions for managers with significant shareholdings can thus counteract the negative influence of environmental uncertainty on digital transformation. Therefore, we hypothesize:

Hypothesis 5a: The negative relationship between environmental uncertainty and digital transformation is weaker in enterprises with higher management shareholding.

### Government subsidies for digital transformation

Government intervention, in the form of subsidies for digital transformation, can play a crucial role in encouraging firms to embrace digital technologies despite the challenges posed by an uncertain environment. By reducing the financial burden associated with digital investments, subsidies can make such initiatives more attractive to risk-averse firms [18]. Moreover, government support signals a commitment to digital development, which can boost confidence and potentially alleviate financial constraints. The availability of subsidies can thus act as a buffer against the negative impact of environmental uncertainty on digital transformation, enabling firms to pursue long-term strategic goals even in turbulent times. Hence, we propose:

Hypothesis 5b: The negative relationship between environmental uncertainty and digital transformation is weaker in enterprises that receive government subsidies for digital transformation.

### Legal institutional environment

A robust legal environment, particularly one that provides strong intellectual property protection, is essential for fostering innovation and encouraging investment in new technologies. In the context of the digital economy, a favorable legal framework can incentivize firms to invest in digital technologies and innovations by safeguarding their intellectual property rights and ensuring a fair competitive environment [40]. Conversely, a weak legal environment, characterized by inadequate protection of intellectual property and high risks of imitation, can deter firms from undertaking digital transformation initiatives. Therefore, we hypothesize:

Hypothesis 5c: The negative relationship between environmental uncertainty and digital transformation is weaker in regions with a more favorable legal environment.

**Fig 1:**
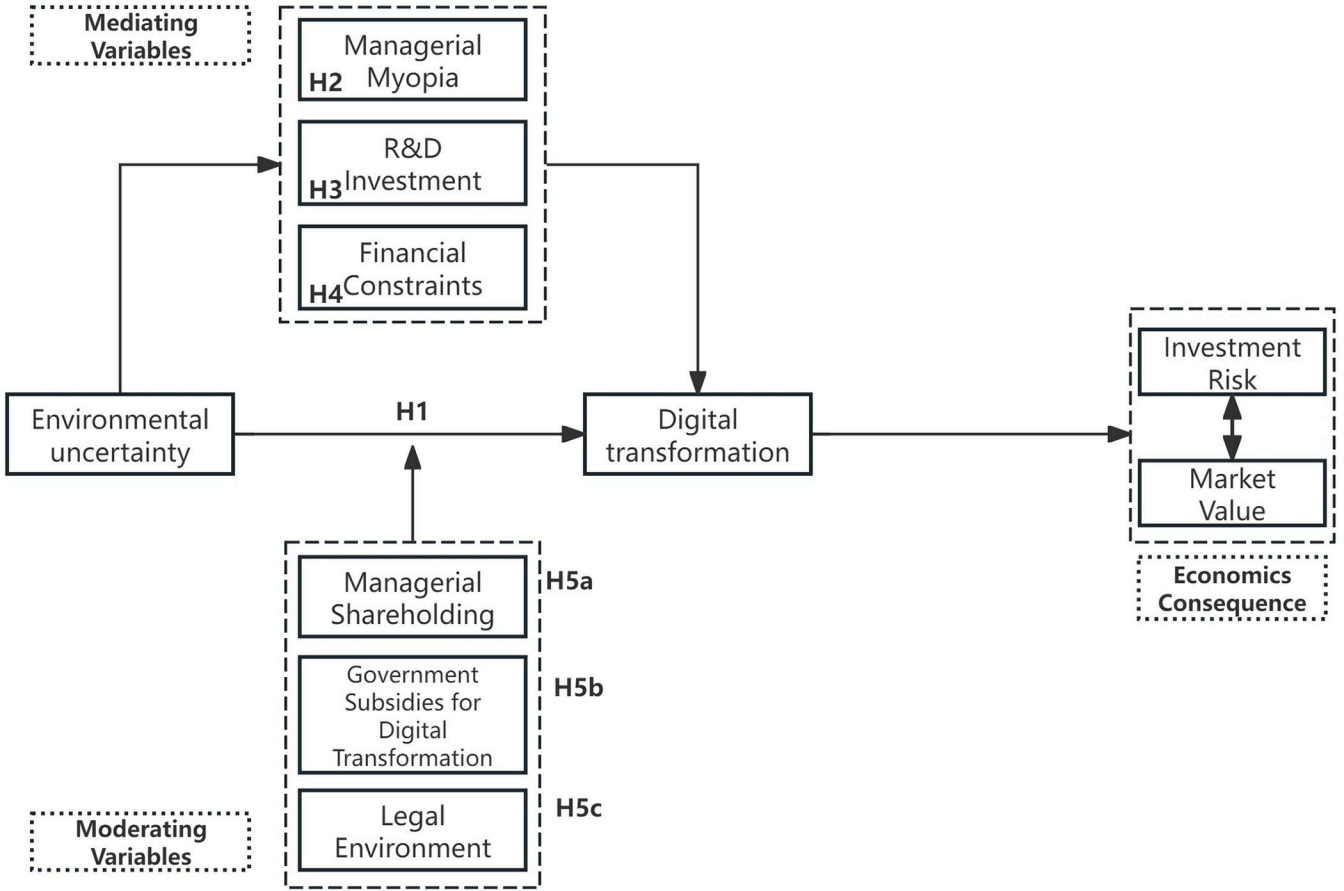
Conceptual framework for the impact of environmental uncertainty on digital transformation: Mediation and moderation effects.

These hypotheses collectively suggest that while environmental uncertainty can pose significant challenges to digital transformation, its negative impact can be mitigated through a combination of internal incentive mechanisms (management shareholding), external financial support (government subsidies), and a conducive legal framework. By leveraging these factors, firms can navigate the complexities of digital transformation and achieve sustainable growth even in the face of uncertainty (Fig 1).

## Methods

### Sample selection and data sources

This study examines A-share listed companies in China from 2013 to 2022, excluding financial institutions (banks, insurance firms), companies marked as ST or *ST, those undergoing IPO during the period, and those with severe missing data. Financial data are sourced from the CSMAR database, consistent with the approach of [41].

### Variable definition and measurement

#### Dependent variable of Digital Transformation (Dt)

This study primarily employs the asset method to measure the degree of digital transformation in enterprises. This is achieved by collecting and consolidating the book value of intangible assets related to digital transformation, as disclosed in the notes to financial statements within the CSMAR database. The degree of digital transformation is then characterized by dividing the sum of these intangible assets by the total assets of the enterprise. This metric reflects the enterprise’s investment in digital capabilities.

In line with [42], this approach is consistent with the understanding that a higher proportion of digital-related intangible assets indicates a greater degree of digital transformation. The intangible assets considered include "software," "management systems," "data platforms," and "information systems," aligning with the Chinese version’s emphasis on these specific categories. This metric reflects the enterprise’s investment in digital capabilities [43–45].

To ensure the robustness of the findings, a robustness test will be conducted using an alternative measurement approach: the text analysis method. This method analyzes the frequency of digital transformation-related terms in corporate annual reports, offering a different perspective on the extent of digital transformation.

By employing both asset-based and text-based measurements, this study aims to provide a comprehensive and reliable assessment of digital transformation within enterprises.

#### Environmental Uncertainty (EU) measurement

Following [17], we measure environmental uncertainty (EU) using the standard deviation of residuals derived from regressing an enterprise’s operating revenue over the past five years on year dummies. The regression model is specified as:

$$Sale=\emptyset_{0}+\emptyset Year+\in$$ (1)

where Sale represents the enterprise’s operating revenue over the five-year period, and Year is a categorical variable with values 1 through 5, representing each year, with 5 being the most recent year. The residuals from this regression capture abnormal fluctuations in operating revenue, after controlling for year-specific effects.

The standard deviation of these residuals is then divided by their mean, yielding an unadjusted indicator of environmental uncertainty. To account for industry-specific factors, this indicator is further normalized by dividing it by the median unadjusted uncertainty of enterprises within the same industry and year. This final industry-adjusted ratio serves as the measure of environmental uncertainty faced by the enterprise, with higher values indicating greater uncertainty.

#### Mediating variables

Management myopia: Measured by real earnings management (REM) level, as in [18], with higher REM indicating greater short-sightedness. R&D Investment is measured by the natural logarithm of R&D expenditure plus one, obtained from financial statement disclosures. This is supported by the extensive literature on R&D and firm performance, as summarized in the meta-analysis by [46]. Financial Constraints is measured by using the FC index, with a higher index indicating greater constraints. This approach aligns with the broader literature on measuring financial constraints, as discussed in [47].

Based on prior studies, we include ownership concentration (SHB), market-to-book ratio (TBQ), firm size (Size), firm age (Age), fixed asset turnover ratio (Tfa), operating cash flow (Cash), and whether the auditing firm is one of the Big Four (Big4). Additionally, industry and year fixed effects are controlled. The inclusion of ownership concentration is consistent with studies on Chinese firms like [12], while the use of market-to-book ratio is supported by classic finance research like [48]. The definitions of these variables are shown in Table 1.

**Table 1:** Variable definitions.

Variable Type	Variable Name	Symbol	Definition
Dependent Variable	Digital Transformation Level	Dt	(Book Value of Digital Intangible Assets / Total Assets) * 100
Independent Variable	Environmental Uncertainty	EU	Coefficient of variation of operating revenue
Mediating Variables	Management Short-sightedness	REM	Real earnings management level (Zhang et al. 2021)
	R&D Investment	RD	Logarithm of R&D expenditure plus one(Kock and Gemünden 2016)
	Financial Constraints	FC	Financial constraint index(Hadlock and Pierce, 2010)
Control Variables	Ownership Concentration	SHB	(Shares held by 2nd-5th largest shareholders / Shares held by largest shareholder)
	Market-to-Book Ratio	TBQ	Market value / (Total assets—Net intangible assets—Net goodwill)
	Enterprise Size	Size	Natural logarithm of total assets at year-end
	Enterprise Age	Age	Logarithm of (Current year—IPO year + 1)
	Fixed Asset Ratio	Tfa	Operating revenue / Average total assets
	Operating Cash Flow	Cash	Net cash flow from operating activities / Operating revenue
	Auditing Firm	Big4	1 if audited by a Big Four firm, otherwise 0
	Industry	Ind	Industry dummy variable
	Year	Year	Year dummy variable

## Model specification

### Baseline regression model

To examine the effect of environmental uncertainty (EU) on digital transformation (Dt), we employ a multiple regression model, drawing on the foundational work of [49,50]. The model is specified as follows:

$$D_{t,it}=\beta_{0}+\beta_{1}{EU}_{it}+\sum \beta_{m}CVS_{it}+\sum {Ind}_{t}+\sum {Year}_{t}+\epsilon_{it}$$ (2)

where subscripts *i* and *t* represent firm and year, respectively. *D_t,it_* denotes the level of digital transformation of firm *i* in year *t*, *EU_it_* represents environmental uncertainty, Control Variables *CVS_it_* encompasses a set of firm-level controls, and Ind represents industry, and Year represents year fixed effects.

### Mediation effect test model

To investigate the mediating role of management short-sightedness, R&D investment, and financial stability in the relationship between EU and DT (Hypothesis 2, 3, and4), we follow the mediation analysis framework proposed by [28,51]. The specific models used to test the indirect effects are:

$${Med}_{it}=\gamma_{0}+\gamma_{1}{EU}_{it}+\sum \gamma_{m}CVS_{it}+\sum {Ind}_{t}+\sum {Year}_{t}+\epsilon_{it}$$ (3)

$$D_{t,it}=\omega_{0}+\omega_{1}{EU}_{it}+\sum \omega_{m}CVS_{it}+\sum {Ind}_{t}+\sum {Year}_{t}+\epsilon_{it}$$ (4)

Where *Med_it_* represents each of the proposed mediating variables: management short-sightedness, R&D investment, or financial stability. All other variables are consistent with the baseline model. Following the guidance of [52], we will estimate the indirect effects using appropriate statistical procedures to assess the significance of the mediating pathways.

## Empirical result

### Descriptive statistical analysis

Table 2 presents the descriptive statistics of the main variables. The average digital transformation level (Dt) of the sample enterprises is 0.580, with a standard deviation of 1.259, a median of 0.207, a maximum value of 11.951, and a minimum value of 0. This indicates that the overall level of digital transformation among listed companies is relatively low, with significant variations between companies. The average environmental uncertainty (EU) is 1.318, with a standard deviation of 1.129, a median of 0.999, a maximum value of 7.895, and a minimum value of 0.111. This suggests that there is considerable variation in the environmental uncertainty faced by the sample enterprises, and the data adequately meet the requirements of this study. Detailed descriptive statistics for other variables are shown in Table 2.

**Table 2:** Descriptive statistic.

Variable Name	N	Mean	Standard Deviation	Median	Minimum	Maximum
Dt	17141	0.580	1.259	0.207	0	11.951
EU	17141	1.328	1.132	1	0.111	7.895
REM	14120	-0.006	0.206	0.014	-0.830	0.698
RD	14907	17.93	2.829	18.23	0	24.622
FC	17141	0.730	0.610	0.554	0.007	3.914
SHB	17141	0.425	0.270	0.419	0.002	0.954
TQB	17141	2.253	1.698	1.761	0.672	42.011
Size	17141	22.600	1.264	22.440	19.680	26.710
Age	17141	2.441	0.566	2.485	1.099	3.401
Tfa	17141	0.204	0.157	0.169	0.001	0.720
Cash	17141	0.098	0.185	0.085	-1.122	0.837
Big4	17141	0.062	0.242	0	0	1

Additionally, the trends in Digital Transformation Level and Environmental Uncertainty Level across the Manufacturing, Services, and Other Sectors industries from 2013 to 2022 (Appendix A in S1 Appendix). The figure provides a visual representation of the variations in these key variables, highlighting the dynamic interplay between digital transformation efforts and the level of environmental uncertainty faced by firms in different sectors.

Table 3 presents the correlation matrix of the variables used in the study, highlighting several important relationships. Digital transformation exhibits a is negative correlation with both environmental uncertainty and firm age, while showing a positivel association with R&D investment and ownership concentration. Environmental uncertainty, on the other hand, is positively correlated with managerial short-sightedness, firm growth, and market-to-book ratio, yet displays negatively associated with R&D investment.

**Table 3:** Correlation matrix.

Variabe	Dt	EU	REM	RD	FC	SHB	TQB	Size	Age	Tfa	Cash	Big4
Dt	1											
EU	-0.018**	1										
REM	-0.087***	0.086***	1									
RD	0.085***	-0.084***	-0.064***	1								
FC	0.100***	0.058***	-0.028***	0.020**	1							
SHB	0.090***	0.026***	-0.070***	-0.179***	0.078***	1						
TQB	0.144***	0.045***	-0.231***	-0.028***	0.057***	0.277***	1					
Size	-0.123***	-0.059***	0.081***	0.243***	-0.070***	-0.866***	-0.369***	1				
Age	-0.085***	-0.006	0.067***	-0.010	-0.157***	-0.379***	-0.191***	0.346***	1			
Tfa	-0.158***	-0.061***	0.020**	-0.056***	-0.056***	-0.110***	-0.121***	0.060***	0.050***	1		
Cash	-0.043***	-0.048***	-0.406***	-0.021**	0.008	-0.072***	0.053***	0.092***	0.003	0.218***	1	
Big4	0.020***	-0.051***	-0.047***	0.125***	-0.029***	-0.246***	-0.056***	0.321***	0.078***	0.025***	0.045***	1

*, **, and *** indicate significance at the 10%, 5%, and 1% levels, respectively.

Moreover, managerial short-sightedness is positively linked with firm size but negatively associated with R&D investment and the presence of a Big4 auditor. In contrast, R&D investment is positively correlation with firm size and a negative association with financial constraints. Lastly, financial constraints show negative associated with firm size and ownership concentration. These interrelationships provide valuable insights that will inform the subsequent analysis and hypothesis testing, helping to clarify the complex dynamics between these variables.

**Fig 2:**
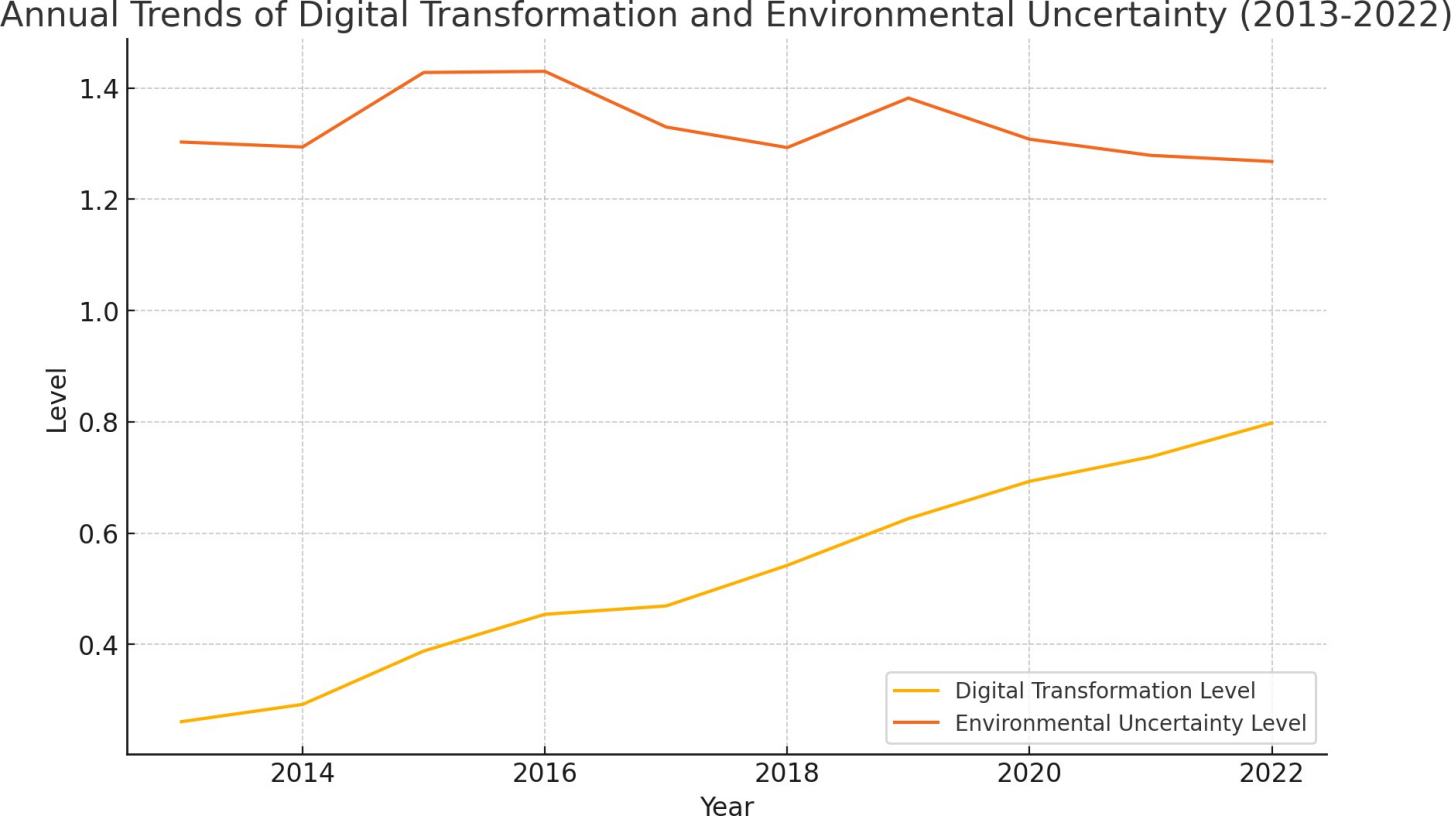
Annual trends of Digital Transformation (D.T.) and Environmental Uncertainty (E.U.) from 2013 to 2022.

The correlation coefficient between the Digital Transformation Level and Environmental Uncertainty Level from 2013 to 2022 is approximately -0.317 (Fig 2). This negative correlation suggests that there is an inverse relationship between these two variables. As the Digital Transformation Level increases, the Environmental Uncertainty Level tends to decrease slightly (Appendix B in S1 Appendix).

### Baseline regression analysis

Table 4 displays the results of Eqs (1) and (2), which examine the impact of environmental uncertainty (EU) on enterprises’ digital transformation (Dt). Across all four model specifications, the findings consistently demonstrate a significant negative relationship between EU and Dt, thereby confirming Hypothesis 1 that environmental uncertainty inhibits digital transformation. This negative correlation remains robust, even after controlling for various firm-specific characteristics, and holds true for both state-owned and privately-owned enterprises.

**Table 4:** Environmental uncertainty (EU) on firms’ digital transformation (Dt) regression result.

Variable	(1)	(2)	(3)	(4)
	Dt	Dt	State-owned	Private
EU	-0.027**	-0.039***	-0.028**	-0.041**
	(-2.125)	(-3.112)	(-2.071)	(-2.431)
SHB		0.079**	0.114**	0.042
		(2.221)	(2.053)	(0.974)
TQB		0.053***	0.067*	0.049***
		(3.315)	(1.870)	(3.401)
Size		-0.053***	-0.023	-0.073***
		(-3.249)	(-1.081)	(-3.343)
Age		-0.005	0.041	-0.043
		(-0.153)	(0.721)	(-1.131)
Tfa		-0.371***	-0.136	-0.567***
		(-3.210)	(-0.924)	(-3.411)
Cash		-0.289***	-0.115	-0.383***
		(-4.012)	(-1.182)	(-3.935)
Big4		0.299***	0.310***	0.287**
		(3.561)	(2.830)	(2.312)
Industry/Year	Yes	Yes	Yes	Yes
cons	-0.032	1.099***	0.275	1.700***
	(-0.876)	(2.945)	(0.553)	(3.541)
N	17141	17141	6674	10027
Adjusted R^2^	0.247	0.265	0.302	0.257

Note: 1) *, **, and *** indicate that the variables are significant at the 10%, 5%, and 1% levels, respectively; 2) the values in parentheses are the cluster-robust t-values.

In addition to this primary finding, the table sheds light on the role of several firm-specific factors in the digital transformation process. Ownership concentration and the use of a Big4 auditor are shown to positively influence digital transformation efforts, whereas factors such as firm size, age, fixed asset turnover ratio, and operating cash flow exert a negative impact. These results emphasize the complex nature of digital transformation, underscoring the need for firms to account for both internal dynamics and external uncertainties when developing their digital strategies.

## Robustness tests and endogeneity tests

### Robustness tests

To assess the robustness of Hypothesis H1, we conduct several sensitivity analyses. First, we replicate the analysis using an alternative measure of digital transformation (Dt) proposed by [53]: the logarithm of the word frequency of digital transformation terms in the Management Discussion and Analysis (MD&A) section of annual reports. Second, we employ the unadjusted environmental uncertainty (EU) as the independent variable, instead of the industry-adjusted measure used in the baseline model. This allows us to examine whether the negative effect of EU on Dt is sensitive to the industry adjustment. Third, we exclude observations from 2020 and 2021 to account for the potential impact of the COVID-19 pandemic on firms’ digital transformation efforts, as suggested by [27].

As shown in Table 5, columns (1), (2), and (3), the coefficient of EU remains significantly negative at the 10%, 1%, and 1% levels, respectively, across all three robustness tests. This consistent result lends further support to Hypothesis H1, indicating that the negative effect of environmental uncertainty on digital transformation is robust to alternative variable definitions and to mitigate the potential influence of the COVID-19 pandemic on firm performance and ensure the robustness of our findings, data from the years 2020 to 2022 are excluded from the sample employed in the subsequent analysis.

**Table 5:** Robustness test results.

Variable	(1)	(2)	(3) Sample Period Adjustment
Dt	Dt	Dt	Dt
EU	-0.022*	-0.398***	-0.037***
	(-1.682)	(-3.300)	(-3.631)
CVS	Yes	Yes	Yes
Industry/Year	Yes	Yes	Yes
cons	-2.277***	1.115***	0.523
	(-5.671)	(2.991)	(1.424)
N	17141	17141	10201
Adjusted R^2^	0.372	0.265	0.273

Note: Sample Period Adjustment: To mitigate potential distortions arising from the COVID-19 pandemic’s impact on business operations, data spanning the years 2020 to 2022 are excluded from subsequent analyses.

### Endogeneity tests

To address potential concerns related to endogeneity, particularly those arising from omitted variables and sample bias, we conduct four robustness checks. As displayed in Table 6, column (1), the coefficient of EU remains significantly negative at the 5% level, indicating that the research findings are robust.

**Table 6:** Endogeneity test results.

Variable	(1) PSM Test	(2) Heckman Test	(3) Lag Effect Test	(4) Region-Time Effect	(5) Industry-Time Effect
EU	-0.053**	-0.032**		-0.038***	-0.039***
	(-3.341)	(-2.382)		(-2.981)	(-3.102)
L.EU			-0.040***		
			(-2.762)		
Imr2		0.123***			
		(3.102)			
CVS	Yes	Yes	Yes	Yes	Yes
Industry/Year	Yes	Yes	Yes	Yes	Yes
Zone×Year				Yes	
Industry×Year					Yes
cons	1.187***	1.072***	1.237***	1.249***	1.103***
	(2.942)	(2.882)	(2.901)	(3.182)	(2.891)
N	7370	17137	13561	17141	17141
Adjusted R^2^	0.264	0.265	0.269	0.280	0.278

*, **, and *** indicate that the variables are significant at the 10%, 5%, and 1% levels, respectively. The values in parentheses are the cluster-robust t-values at the enterprise level.

To tackle potential sample bias, we apply propensity score matching (PSM). We generatea dummy variable (EU_D) to indicate whether a firm’s environmental uncertainty (EU) exceeds the industry-year average. Using covariates such as return on assets, market-to-book ratio, ownership concentration, firm size, age, sales growth, fixed asset turnover, operating cash flow, management ownership, and auditor type, we perform 1:1 nearest-neighbor matching with replacement. The results, shown in column (1) of Table 6, confirm that EU has a negative and significant effect on digital transformation(DT) at the 5% level, lending support Hypothesis H1.

Building on [9], we employ the average EU of other firms in the same industry and year (excluding the focal firm) as an instrumental variable. This, along with other control variables, is incorporated into the first stage of the Heckman model. The inverse Mills ratio (IMR2) obtained from this stage is then added to the second-stage regression alongside the original model. The findings, as presented in column (2) of Table 6, show that the coefficient of EU remains negative and significant at the 5% level, further validating Hypothesis H1.

To account for potential time lags and omitted variable bias, we introduce a one-period lag of EU into the model. The results, presented in column (3) of Table 6, indicate that the lagged effect of EU (L.EU) is negative and significant at the 1% level, providing further evidence of robustness of Hypothesis H1.

Lastly, to address unobserved heterogeneity at both the region and industry levels, we incorporate region-time and industry-time fixed effects into the model. As reflected in columns (4) and (5) of Table 6, the results consistently show a negative and significant effect of EU on DT at the 1% level, freinforcing the robustness of our findings.

we further applied the ITCV method as proposed by [54] to reexamine the potential for omitted variable bias. Upon conducting the test, we found that the ITCV value for the baseline model was 0.596. The maximum correlation coefficient between the control variables, the independent variable (environmental uncertainty), and the dependent variable (digital transformation) was 0.144 the highest correlation coefficient being between Tobin’s Q (TQB) and digital transformation (Dt). This indicates that omitted variable bias does not significantly impact the stability of the model.

### Mediation mechanism test

The results of the mediation analyses, presented in Table 7, offer compelling evidence that environmental uncertainty indirectly inhibits digital transformation through the interaction of several key mechanisms. The positive and significant coefficient of EU in column (1), alongside the negative and significant coefficient of REM in column (2), confirms that heightened environmental uncertainty fosters managerial myopia, which subsequently impedes digital transformation. This finding supports Hypothesis 2, underscoring the harmful influence of short-term thinking when navigating the complexities of digital initiatives in uncertain environments.

**Table 7:** Mediation mechanism test results.

Variable	(1) REM	(2) Dt	(3) RD	(4) Dt	(5) FC	(6) Dt
EU	0.017***		-0.158***		0.009***	
	(5.812)		(-5.142)		(2.951)	
REM		-0.494***				
		(-5.231)				
RD				0.033***		
				(6.412)		
FC						-0.429***
						(-3.612)
CVS	Yes	Yes	Yes	Yes	Yes	Yes
Industry/Year	Yes	Yes	Yes	Yes	Yes	Yes
cons	1.103***	0.429	-3.240***	1.417***	0.807***	3.109***
	(2.891)	(1.282)	(-3.571)	(3.241)	(18.501)	(4.561)
N	14120	14120	14907	14907	17414	17141
Adjusted R^2^	0.244	0.282	0.279	0.266	0.300	0.264

Additionally, the analysis highlights the mediating role of R&D investment. The negative and significant coefficient of EU in column (3) demonstrates that environmental uncertainty discourages R&D investment. Conversely, the positive and significant coefficient of RD in column (4) shows that increased R&D investment promotes digital transformation. These results lend support to Hypothesis 3, suggesting that the reluctance to invest in R&D during periods of uncertainty indirectly hampers a firm’s progress in digitalization.

Finally, the evidence in columns (5) and (6) confirms the mediating role of financial constraints. The positive and significant coefficient of EU in column (5), together with the negative coefficient of FC in column (6), illustrates that environmental uncertainty exacerbates financial constraints, which in turn hinder digital transformation efforts. This supports Hypothesis 4, establishing financial constraints as a crucial mechanism through which environmental uncertainty negatively affects digital transformation.

In sum, Table 7 provides a detailed perspective on how environmental uncertainty indirectly obstructs digital transformation. It reveals the interconnected roles of managerial myopia, R&D investment, and financial constraints, demonstrating how these factors collectively create a challenging environment for firms striving to adopt digital technologies in the face of uncertainty.

### Mitigating the negative impact of environmental uncertainty on digital transformation

The analysis conducted reveals that environmental uncertainty hampers digital transformation by promoting managerial myopia, reducing the intensity of R&D investment intensity, and increasing financial constraints. However, in practice, firms may be able to counteract these negative effects through the implementation of effective internal incentive and operating within a supportive legal environment. Specifically, this study explores whether higher levels of managerial shareholding, government subsidies aimed at digital transformation, and a favorable legal environment can mitigate the adverse effects of environmental uncertainty.

By examining these potential moderating mechanisms, our research seeks to offer practical insights for firms attempting to pursue digital transformation amidst uncertainty. Furthermore, this study aims to inform targeted policy interventions that governments can implement to support and accelerate the digital transformation process.

Table 8 presents empirical evidence regarding the economic consequences of environmental uncertainty (EU) on firms’ digital transformation (Dt) and subsequent performance. The findings demonstrate a significant negative effect of EU on Dt, supporting the hypothesized inhibitory relationship. These results suggest that increased uncertainty discourages firms from investing in digital technologies, which can, in turn, have harmful implications for their long-term growth and resilience.

**Table 8:** Result of economic consequences of environmental uncertainty (EU) on firms’ digital transformation (Dt).

Variable	(1) Dt	(2) Risk	(3) MV
EU	-0.039*** (-3.112)		
Dt		0.002*** (3.071)	0.024*** (3.770)
CVS	Yes	Yes	Yes
Industry/Year	Yes	Yes	Yes
_cons	1.099*** (2.945)	0.120*** (12.551)	4.800*** (28.731)
N	17141	17001	17141
Adjusted R^2^	0.265	0.122	0.861

*, **, and *** indicate significance at the 10%, 5%, and 1% levels, respectively.

Previous analysis indicates that environmental uncertainty hinders digital transformation by fostering managerial short-sightedness, reducing R&D investment, and increasing financial constraints. To address this, we explore the potential mitigating effects of effective internal incentive mechanisms and a favorable legal environment. Specifically, we examine whether increased management shareholding, digital transformation subsidies, and a conducive legal environment can alleviate the negative impact of uncertainty on digital transformation, offering insights for both enterprises and policymakers. These findings underscore the importance of digital transformation as a strategic imperative for firms operating in uncertain environments, and highlight the potential economic costs of delaying or neglecting digital initiatives in the face of uncertainty.

### Management shareholding

Building on agency theory, we hypothesize that increasing management shareholding can align managerial interests more closely with those of the firm, thereby mitigating short-sighted behavior that can arise from heightened environmental uncertainty [8]. This alignment increases the personal cost of short-sighted decisions for managers, discouraging them from prioritizing immediate gains over long-term value creation, particularly in the context of risky digital transformation initiatives. To test this hypothesis of 5a, we introduce an interaction term (EU × M-share) in model, where M-share indicates whether management shareholding exceeds the sample median.

The results in Table 9, columns (1)-(3), lend support this hypothesis. The positive and significant coefficient of EU × M-share in column (1) suggests that increased management shareholding indeed weakens the negative effect of environmental uncertainty on digital transformation. This mitigating effect is further substantiated by the negative and significant coefficient of EU in column (2) for firms with low management shareholding, in contrast the non-significant coefficient in column (3) for firms with high management shareholding.

**Table 9:** Effects of management shareholding and digital subsidies on the relationship between environmental uncertainty and digital transformation.

Variable	(1) Full Sample	(2) Low Management Shareholding	(3) High Management Shareholding	(4) Full Sample		(5) No Digital Subsidy	(6) With Digital Subsidy
EU	-0.056*** (-4.791)	-0.041*** (-4.092)	-0.034 (-1.521)	-0.053*** (-4.620)	-0.035*** (-3.361)		-0.057 (-1.082)
EU×M-share	0.035* (1.883)						
EU×Sub				0.143*** (3.693)			
CVS	Yes	Yes	Yes	Yes		Yes	Yes
Industry/Year	Yes	Yes	Yes	Yes		Yes	Yes
_cons	1.067*** (2.873)	0.538 (1.064)	1.972*** (3.201)	1.146*** (3.062)		0.760** (2.281)	4.993*** (3.172)
N	17141	8082	9059	17141		15126	2015
Adjusted R^2^	0.265	0.246	0.269	0.269		0.251	0.270

*, **, and *** indicate significance at the 10%, 5%, and 1% levels, respectively.

### Government subsidies for digital transformation

Government subsidies targeted at digital transformation can serve as a crucial policy instrument to encourage firms to embrace digital technologies, even amidst uncertainty. By reducing the financial burden associated with digital investments, subsidies can make such initiatives more appealing to risk-averse firms. Moreover, government support signals a commitment to digital development, boosting confidence and potentially easing financial constraints.

To test this proposition in our hypothesis 5b, we introduce an interaction term (EU × Sub) in model (1), where Sub indicates whether a firm received digital transformation subsidies. The results in Table 9, columns (4)-(6), reveal a positive and significant coefficient for EU × Sub, suggesting that subsidies effectively mitigate the negative impact of environmental uncertainty. This observation is further supported by the negative and significant coefficient of EU for firms without subsidies, compared to the non-significant coefficient for firms with subsidies.

### Legal institutional environment

A sound legal environment, particularly one characterized by robust intellectual property protection, is fundamental for fostering innovation. In the context of the digital economy, strong legal frameworks can incentivize firms to invest in digital technologies and innovations. Conversely, a weak legal environment, marked by inadequate protection of intellectual property and high risks of imitation, can discourage firms from undertaking digital transformation initiatives.

To examine this dynamic of hypothesis 5c, we introduce an interaction term (EU × Env) in model (1), where Env represents the quality of the legal environment in the firm’s location. The results in Table 10, columns (1)-(3), demonstrate a positive and significant coefficient for EU × Env, indicating that a favorable legal environment mitigates the negative effect of environmental uncertainty. This is further corroborated by the negative and significant coefficient of EU in regions with a weaker legal environment and a less significant coefficient in regions with a stronger legal environment.

**Table 10:** Effect of legal environment on the relationship between environmental uncertainty and digital transformation.

Variable	(1) Full Sample	(2) Poor Legal Environment	(3) Good Legal Environment
EU	-0.064***	-0.039***	-0.033*
	(-5.190)	(-3.401)	(-1.671)
EU×Env	0.052***		
	(2.901)		
CVS	Yes	Yes	Yes
Industry/Year FE	Yes	Yes	Yes
cons	1.104***	1.174***	1.014*
	(2.963)	(2.762)	(1.961)
N	17141	8111	9030
Adjusted R2	0.266	0.237	0.277

*, **, and *** indicate significance at the 10%, 5%, and 1% levels, respectively.

Overall, these findings highlight the importance of internal incentive mechanisms and external institutional support in mitigating the adverse effects of environmental uncertainty on digital transformation.

## Discussion

This study contributes to the growing body of literature on the intricate relationship between external uncertainties and digital transformation. Our findings reveal that environmental instability significantly hampers digital transformation efforts, primarily by promoting managerial short-termism, reducing R&D investment, and exacerbating financial constraints. These results are consistent with risk aversion theory [11] and real options theory [21], which suggest that firms tend to become more cautious in their investment decisions when faced with heightened uncertainty. We extend this understanding by providing empirical evidence of the specific mechanisms through which environmental uncertainty influences digital transformation decisions.

Furthermore, our study highlights the critical role that environmental stability plays in facilitating long-term investment in digital technologies. This finding aligns with previous studies emphasizing the importance of a stable and predictable environment for firms to engage in strategic long-term planning and innovation [36,54]. By demonstrating the detrimental effects of environmental uncertainty on digital transformation, our study underscores the need for proactive measures to mitigate these negative impacts.

In line with agency theory [8], our analysis reveals that increasing management shareholding can mitigate the negative effects of environmental uncertainty on digital transformation. This finding corroborates existing literature, which suggests that aligning the interests of managers and shareholders can reduce opportunistic behavior and promote long-term value creation, particularly in the context of digital transformation [25]. Additionally, we find that government subsidies for digital transformation can help firms overcome financial constraints in uncertain environments, echoing prior research on the importance of government support in fostering digital innovation [55].

Our study also highlights the crucial role of a supportive legal environment in promoting digital transformation amidst uncertainty. This finding resonates with previous research emphasizing the importance of strong legal frameworks, particularly those protecting intellectual property rights, in encouraging firms to invest in digital technologies and innovations [53].

Our study makes several contributions to the existing literature. First, we adopt a broader and more nuanced conceptualization of environmental instability, encompassing a wider array of external shocks beyond economic policy uncertainty (EPU). This broader scope allows us to capture a more comprehensive understanding of how firms navigate and adapt to multifaceted uncertainties in their external environment. Second, we provide empirical evidence of the specific mechanisms through which environmental instability obstructs digital transformation. This contributes to a deeper understanding of the challenges firms face when pursuing digital initiatives in uncertain times. Third, our study introduces the moderating roles of management shareholding, government support, and legal environments, which can significantly mitigate the negative effects of external instability. This offers valuable insights for both enterprises and policymakers seeking to promote digital transformation in dynamic environments.

## Conclusion

This study provides new insights into the relationship between environmental uncertainty and digital transformation by empirically analyzing a sample of Chinese A-share listed companies from 2013 to 2022. Our results reveal that environmental instability significantly hampers digital transformation efforts, primarily by promoting managerial short-termism, reducing R&D investment, and exacerbating financial constraints. These findings contribute to the academic literature by advancing our understanding of how external uncertainty shapes organizational digital strategies and decision-making. Specifically, this research highlights the critical role that environmental stability plays in facilitating long-term investment in digital technologies.

From a managerial perspective, our study underscores the importance of adopting proactive strategies to mitigate the adverse effects of environmental uncertainty on digital transformation. By increasing management shareholding, companies can align leadership interests with long-term organizational goals, thus reducing the tendency toward short-termism. Additionally, digital transformation subsidies can help firms overcome financial constraints, particularly in volatile environments. These practical strategies offer managers concrete tools to navigate uncertainty and foster sustainable digital innovation.

In terms of public policy, our findings suggest that governments can play an instrumental role in supporting firms’ digital transformation efforts under uncertain conditions. By offering targeted subsidies and fostering a supportive legal and regulatory environment, policymakers can alleviate the financial and regulatory burdens that inhibit digital investments. This has broader implications for national competitiveness and technological advancement, as enabling more firms to engage in digital transformation will lead to more resilient, innovative, and adaptable economies.

In sum, this study contributes to both theory and practice by identifying key mechanisms to overcome the challenges posed by environmental uncertainty, while also offering policy recommendations that can facilitate digital transformation at a broader societal level.

### Limitations and future research

While our study focuses on the historical impact of environmental instability on digital transformation, it offers a crucial foundation for future research to explore the predictive potential of these findings. Subsequent studies could employ more advanced data and forecasting techniques to develop models that predict how environmental instability might influence digital transformation across different scenarios and time horizons.

Additionally, future research should delve deeper into the mechanisms underlying these effects, potentially examining the roles of organizational culture, leadership dynamics, and external stakeholder pressures. Such investigations would allow for a more comprehensive exploration of the complex interplay between external uncertainties and digital transformation strategies, shedding light on the nuanced ways in which firms adapt and innovate in an increasingly uncertain world.

## Supporting information

S1 Appendix Contains both the Appendix A and B. (DOCX)
