# Supplementary material for: Navigating uncertainty: The impact of environmental instability on enterprise digital transformation
Source: PLoS One. 2024 Dec 5;19(12):e0314688. doi: 10.1371/journal.pone.0314688 (PMC11620587; doi:10.1371/journal.pone.0314688)
Supplement: S1 Appendix — (DOCX) [file pone.0314688.s001.docx]

Appendix A Digital Transformation v.s. Environmental Uncertainty by Industry Sectors

| Year | Industry | Digital Transformation Level | Environmental Uncertainty Level |
| --- | --- | --- | --- |
| 2013 | Manufacturing | 0.230 | 1.231 |
| 2013 | Services | 0.761 | 1.523 |
| 2013 | Other Sectors | 0.116 | 1.370 |
| 2014 | Manufacturing | 0.298 | 1.235 |
| 2014 | Services | 0.592 | 1.269 |
| 2014 | Other Sectors | 0.155 | 1.405 |
| 2015 | Manufacturing | 0.335 | 1.356 |
| 2015 | Services | 0.994 | 1.424 |
| 2015 | Other Sectors | 0.177 | 1.613 |
| 2016 | Manufacturing | 0.362 | 1.375 |
| 2016 | Services | 1.108 | 1.498 |
| 2016 | Other Sectors | 0.230 | 1.538 |
| 2017 | Manufacturing | 0.384 | 1.289 |
| 2017 | Services | 1.098 | 1.492 |
| 2017 | Other Sectors | 0.248 | 1.326 |
| 2018 | Manufacturing | 0.429 | 1.283 |
| 2018 | Services | 1.244 | 1.364 |
| 2018 | Other Sectors | 0.277 | 1.263 |
| 2019 | Manufacturing | 0.481 | 1.362 |
| 2019 | Services | 1.480 | 1.380 |
| 2019 | Other Sectors | 0.298 | 1.447 |
| 2020 | Manufacturing | 0.518 | 1.310 |
| 2020 | Services | 1.624 | 1.250 |
| 2020 | Other Sectors | 0.353 | 1.359 |
| 2021 | Manufacturing | 0.532 | 1.244 |
| 2021 | Services | 1.804 | 1.319 |
| 2021 | Other Sectors | 0.373 | 1.355 |
| 2022 | Manufacturing | 0.549 | 1.255 |
| 2022 | Services | 2.102 | 1.249 |
| 2022 | Other Sectors | 0.400 | 1.333 |

Appendix B Changes by Digital transformation v.s. Environmental Uncertainty

| Year | Digital Transformation Level（Annual average） | Environmental Uncertainty Level（Annual average） |
| --- | --- | --- |
| 2013 | 0.261 | 1.303 |
| 2014 | 0.292 | 1.294 |
| 2015 | 0.388 | 1.428 |
| 2016 | 0.454 | 1.430 |
| 2017 | 0.469 | 1.330 |
| 2018 | 0.542 | 1.293 |
| 2019 | 0.626 | 1.382 |
| 2020 | 0.693 | 1.308 |
| 2021 | 0.737 | 1.279 |
| 2022 | 0.798 | 1.268 |
